# Improving Bovine Tuberculosis Surveillance Through Risk-Based Prioritization of Slaughterhouse-Triggered Trace-Back Investigations

**DOI:** 10.3390/ani16081224

**Published:** 2026-04-16

**Authors:** Luiz Felipe Crispim Lourenço, Ricardo Evandro Mendes

**Affiliations:** 1 Companhia de Desenvolvimento Agrícola de Santa Catarina (CIDASC), Florianópolis 88034-001, SC, Brazil; 2 Athens Veterinary Diagnostic Laboratory (AVDL), Department of Pathology, College of Veterinary Medicine, University of Georgia, Athens, GA 30602, USA

**Keywords:** risk-based surveillance, cattle, social network risk analysis, disease eradication, official veterinary services

## Abstract

Slaughterhouse detection of lesions compatible with bovine tuberculosis represents a key passive surveillance component in Santa Catarina, Brazil, yet subsequent trace-back investigations often fail to identify infected farms. This study developed a quantitative framework to prioritize epidemiological investigations by estimating the probability of infection associated with each farm connected to PCR-confirmed cases. Using official movement records and historical diagnostic data, we reconstructed the lifetime contact networks of slaughtered cattle presenting confirmed *Mycobacterium bovis* lesions *(n* = 502). For each sentinel animal–farm interaction (*n* = 1452), infection probability was estimated through a non-homogeneous Poisson process incorporating exposure duration and the time-weighted average herd size as determinants of infectious pressure. After evaluating stochastic variability through Monte Carlo simulation, a deterministic model using the mean infectious-pressure parameter was applied to classify farms into high-, medium-, and low-risk categories. Model performance was assessed using validated field diagnostic outcomes within a three-year temporal window. High-risk farms represented most validated contacts (58%) and demonstrated a relative risk of 3.48 compared with lower-risk category. These findings indicate that a standardized risk-based classification can substantially improve the prioritization of trace-back investigations, offering a practical decision-support tool to enhance bovine tuberculosis surveillance and contribute to eradication strategies in Santa Catarina.

## Simple Summary

Bovine tuberculosis is a long-lasting infectious disease that affects cattle and can cause major economic and public health concerns. In the state of Santa Catarina, Brazil, slaughterhouses routinely examine cattle for suspicious lesions, and when a lesion is confirmed as tuberculosis, veterinary services must investigate all farms that had epidemiological contact with that animal. However, many of these investigations do not uncover an active infection, partly because there is no standardized way to decide which farms are most likely to be involved in disease transmission. This study created a risk-classification system to help guide these investigations. By using official animal-movement records and information about the amount of time animals (*n* = 502) spent on each farm and the number of cattle they contacted, we calculated the likelihood that each farm was part of a transmission chain. The model was then compared with real field comparative intradermal testing results to determine how well the risk scores predicted true infections. Our findings show that this approach can help veterinary authorities focus their efforts on farms with the highest probability of infection, improving the efficiency of surveillance and supporting the long-term goal of eliminating bovine tuberculosis in the region.

## 1. Introduction

Bovine tuberculosis represents a critical global challenge due to its simultaneous impact on public health and livestock economies [1,2]. As a zoonotic disease, it is primarily transmitted to humans through the consumption of raw dairy products or direct occupational contact with infected animals [3,4]. However, the true scale of this threat is often underestimated because many infected bovines remain asymptomatic, allowing the pathogen to circulate undetected within the dairy chain. In some endemic regions, molecular testing has identified the presence of the pathogen in nearly half of raw milk samples, highlighting a significant failure in surveillance and milk hygiene.

Economically, the disease serves as a major drain on agricultural productivity. Infected animals suffer from compromised health and welfare, and nearly half of those identified with the disease exhibit visible lesions during post-mortem inspections. These findings result in the partial or total condemnation of carcasses, rendering them unfit for human consumption and causing direct financial losses to producers. In regions across Latin America, Africa, and Asia, these factors combine to hinder livestock development and sustain a cycle of transmission between humans and cattle. Addressing this dual burden requires an integrated approach that bridges the gap between veterinary and medical health systems to achieve effective disease control [1,4].

In 2001, Brazil established the National Program for the Control and Eradication of Brucellosis and Animal Tuberculosis (PNCEBT) [5], which introduced a risk-based classification for states according to disease prevalence [6]. For the state of Santa Catarina, this baseline status was defined in 2009 through a comprehensive epidemiological survey conducted by Veloso et al. [7]. The results led to the state’s classification as ‘Category A’—a status indicating negligible or very low risk, contingent upon periodic audits of the official veterinary services [8]. Consequently, Santa Catarina shifted its strategic focus from disease control to full eradication.

This eradication strategy relies on a strict ‘test-and-cull’ protocol as the primary sanitization method. Upon the identification of any number of infected animals, the farm is placed under movement restrictions. The herd then undergoes successive rounds of testing including all bovines in the herd and culling of all positive animals. A mandatory interval of at least 15 days is observed following the removal of the last infected animal before a new round begins, and the quarantine is only lifted after the herd achieves two consecutive negative results.

The gold standard diagnostic tools defined by the PNCEBT are the intradermal tuberculin tests, which include the Single Cervical Test (SCT), the Caudal Fold Test (CFT), and the Comparative Cervical Test (CCT) [6]. However, in the state of Santa Catarina, the CCT is the most widely used test, as it is the only test officially recognized for triggering government indemnity following animal culling. Despite their regulatory status, these tests present surveillance limitations due to the high variability of sensitivity and specificity in field conditions [9,10]. This instability is often driven by anergy in chronically infected animals or the temporary suppression of immune responses, which can mask the presence of the pathogen. Additionally, the need for mandatory intervals between tests to avoid desensitization, coupled with potential cross-reactivity, often necessitates repeat testing to confirm a herd’s status, complicating the speed and accuracy of disease eradication efforts.

Surveillance for bovine tuberculosis in Santa Catarina is structured as a multi-layered framework. Preventive measures include the mandatory testing of breeding animals prior to movement and triennial testing for all dairy herds. Complementing these active measures, passive surveillance at slaughterhouses serves as a critical safety net: suspicious lesions identified during routine inspection are collected and submitted for molecular confirmation via PCR (Polymerase Chain Reaction). To detect *M. bovis* using PCR, suspicious lesions are first ground up and treated to release the bacterial DNA from the tough, waxy cells. This DNA is then purified and amplified using PCR to target specific genetic markers unique to the bacteria. This process allows for a rapid diagnosis in days rather than months, though it can still be limited by the amount of intact DNA present in the tissue sample [9,11]. However, detection of a confirmed lesion does not always equate to the identification of a field outbreak. When an outbreak is detected—whether through field testing or slaughterhouse feedback—veterinary services initiate trace-back and trace-forward investigations across all epidemiologically linked farms. In practice, a substantial proportion of these investigations do not lead to the identification of secondary outbreaks. This suggests that while the initial slaughterhouse PCR results are reliable, the subsequent tracing through complex movement networks often results in cold cases. Compounding this challenge is the absence of optimized, quantitative criteria for prioritizing which transit-related farms should be investigated first, resulting in the allocation of limited resources without standardized risk-assessment tools. In practical terms, rather than utilizing a quantitative hierarchy, veterinarians prioritize farms based on empirical perceptions of risk. While grounded in professional judgment, this approach lacks standardization and is frequently influenced by the immediate complexity of the movement network and available resources.

To address these limitations, this study developed a stochastic model designed to evaluate the risk associated with movement chains originating from slaughterhouse-detected lesions. By integrating cattle transit records with an infection-probability algorithm, the model classified potential contact farms into risk categories based on their likelihood of being involved in the infection chain. This approach provides a quantitative tool to standardize the prioritization of trace-back investigations, which are currently dependent on discretionary field criteria. In this work, we describe the development of this classification model and validate its performance by comparing assigned risk levels with documented field outcomes in the state of Santa Catarina. Our objective is to demonstrate how a standardized risk-classification system can optimize the investigation from slaughterhouse PCR-positive cases, improving the efficiency of official veterinary services aiming bovine tuberculosis eradication.

## 2. Materials and Methods

### 2.1. Data Retrieval

The study was based on official records provided by the Santa Catarina Agricultural Defense Management System (Sigen+, developed by CIASC (Centro de Informática e Automação do Estado de Santa Catarina, Florianópolis, Brazil)), the official database maintained by the Santa Catarina Integrated Agricultural Development Company (CIDASC, Florianópolis, Brazil) [12]. Data collection focused on suspicious granulomatous lesions identified during routine post-mortem inspections in slaughterhouses across the state between 2021 and 2025, which were subsequently confirmed as *Mycobacterium bovis* through Polymerase Chain Reaction (PCR). Cases were selected based on data completeness and traceability; meaning, cases with incomplete data were not included. Since Santa Catarina maintains individual bovine traceability through official ear tags in accordance with the SISBOV [13] standard, it was possible to retrieve the complete life history of each sentinel animal. In this study, a sentinel animal was defined as a slaughtered bovine presenting a PCR-confirmed *M. bovis* lesion that served as the index case for reconstructing its historical movement chain and all associated epidemiological contact farms. The extracted dataset included individual movement records (2009–2025) and information characteristics of contact holdings, including field tests of all bovine contacts (2015–2025), when available. Furthermore, for each holding, the Time-Weighted Average contact herd size (*H*) was calculated by reconstructing the daily co-location pattern between the sentinel animal and all other bovines present on the farm. Using official entry and exit dates for every individual, we determined the number of animals that overlapped with the sentinel animal on each day of the exposure interval. The final value of *H* corresponds to the mean number of overlapping animals, weighted by the duration of daily contact, thus reflecting both herd size and the temporal distribution of direct exposure events. All data were processed within the Sigen+ database environment, de-identified to ensure data privacy, and exported in Apache Parquet format to facilitate high-performance computational analysis. From this stage onward, the processed datasets were available as Supplementary Material in a public GitHub repository (see Supplementary Materials section for details).

### 2.2. Modeling

To estimate the probability of infection for a sentinel animal during farm-level interactions, we employed a non-homogeneous Poisson process framework. This model assumes that infection events occur stochastically over time, where the probability of the animal remaining uninfected decays exponentially as exposure accumulates [4].

The cumulative risk (P) is modeled as the complement of the probability that no transmission occurs. This framework naturally accounts for risk saturation, where increasing exposure duration or population density leads to a non-linear approach toward a near-certain infection event (P =1), reflecting the biological reality of diminishing returns in infectious pressure. In this context, the risk is formulated as

$$P=1-e^{-\lambda \cdot T\cdot \ln\left(H\right)}$$

where

- P represents the estimated probability of infection;
- λ  is the stochastic risk parameter representing the infectious pressure per day of contact;
- T is the exposure duration;
- H is the time-weighted average of the contact herd size during the exposure duration T.

In this framework, the stochastic risk parameter (λ) and the exposure duration (T) function as linear escalators of risk, representing the principle that the probability of infection accumulates proportionally over time under a constant infectious pressure. Conversely, contact intensity is incorporated as the natural logarithm of *H*, which is calculated by normalizing cumulative daily animal contacts over the total exposure period. This choice is grounded in the principle of density-dependent saturation, assuming that the incremental risk of infection per additional bovine contact diminishes as herd size increases, following a non-linear pressure curve rather than an infinite linear progression [14]. While factors such as production system and specific animal density may influence the saturation curve [7], these variables are not currently recorded in the state database. Consequently, the logarithmic transformation of H serves as a generalized proxy for density-dependent saturation based on the available data. Furthermore, a critical algorithmic constraint was implemented to ensure biological consistency: in instances where the contact intensity was negligible (h ≤ 1), indicating a lack of effective interaction with other bovines, the infection probability was strictly defined as zero. This adjustment prevents the model from generating mathematical artifacts or negative values through the logarithmic transformation of transient or non-existent contacts, ensuring that the resulting risk scores remain biologically plausible and focused on effective exposure events. This threshold reflects the nature of bovine tuberculosis as a primarily density-dependent disease, where transmission relies on close animal-to-animal interaction. Since environmental persistence of *M. bovis* contributes minimally to new infections compared to direct aerosol exposure, a herd size below one effectively nullifies the probability of maintaining a transmission chain [14,15].

A fundamental premise of this modeling approach is its treatment of transmission as a bidirectional risk interface. Unlike traditional mechanistic models designed to identify the source of an index case [16], this framework considers the probability (P) as a shared risk of exposure between the sentinel animal and the contact holding. We assume that the pathogen could have moved to the farm from either direction: either the sentinel animal was infected by the local herd or it may have introduced the infection into a previously clean farm. By remaining agnostic to the exact direction of transmission, the model prioritizes the classification of risk at the contact points where a bridge for *M. bovis* may have been established. This ensures that the resulting risk class reflects the potential for an active field outbreak at each location and time of contact.

To address the inherent biological uncertainty of the daily infectious pressure (λ), we initially implemented a stochastic modeling approach. The parameter λ was represented by a PERT distribution [17], a specialized Beta distribution defined by a minimum of 10 × 10^−5^, a mode of 2.7 × 10^−5^, and a maximum of 8.0 × 10^−5^ [7].

### 2.3. Model Validation

We then executed a Monte Carlo simulation with 5000 iterations per contact to propagate this uncertainty through the risk calculation. However, an analysis of the resulting Coefficient of Variation (CV) demonstrated that the model’s output was overwhelmingly dominated by the structural variables: exposure duration (T) and the log-transformed contact herd size (ln(H)). Additionally, a sensitivity analysis using class retention rates and Cohen’s Kappa was performed to evaluate the stability of the risk classification under stochastic pressure. Since the stochasticity of λ contributed marginally to the final risk ranking compared to the influence of time and population density, we determined that a deterministic simplification was sufficiently sensitive for practical surveillance. Consequently, the subsequent risk classification and field validation stages were conducted using a deterministic λ  based on the PERT distribution mean (λ ≈ 3.17 × 10^−5^).

To calibrate the risk classification and ensure the mathematical integrity of the parameters, the analysis was restricted to one-to-one sentinel–farm relationships. By isolating animals with single-farm histories, we prevented the dilution of risk across multiple contact events, following the anchoring principle [18,19] to capture the maximum potential infectious pressure without the confounding effects of diverse contact farms. These thresholds—defining high (p > 0.95), medium (0.50 ≤ p ≤ 0.95), and low risk (p < 0.50)—were then applied to the P values estimated for all farms within the complete movement dataset.

The model’s predictive performance was validated against field diagnostic records, using a classification logic designed to account for variations in surveillance intensity and data completeness. A farm was classified as positive upon the detection of any infected animal within the herd during a diagnostic round. However, to mitigate the risk of false negatives, a negative status was only assigned to farms where the sampling effort met specific statistical confidence thresholds. Diagnostic events were aggregated into pools or rounds, defined as tests occurring within a 90-day window, to represent a single epidemiological snapshot of the holding. Negative results within these rounds were categorized as Gold Standard if the testing coverage reached at least 80% of the total herd size (n/N = 0.80) or if at least 60 animals were tested (n ≥60). These thresholds are statistically grounded in the ability to detect at least one positive animal with 95% confidence, assuming a minimum within-herd prevalence of 5% in large populations. In instances where these criteria were not met, the status was still considered acceptable if the sampling fraction reached at least 30% of the herd (n/N = 0.30) or a minimum of 20 animals were tested (n ≥ 20), a threshold sufficient to detect a 15% within-herd prevalence with 95% confidence. Farms failing to meet these minimum requirements were classified as Under-sampled. During this stage, we measured and validated the Positive Predictive Values (PPV) and Negative Predictive Values (NPV) for each of the model’s risk classes (e.g., High Risk vs. confirmed field infection). Furthermore, these metrics were compared against a baseline of “pure” slaughterhouse surveillance, defined as the total set of identified contacts versus confirmed field outbreaks, to evaluate the incremental gain in precision provided by the stochastic risk framework. This methodology ensures that negative classifications are anchored in evidence of absence, providing a robust ground-truth for evaluating the model’s performance against traditional epidemiological tracing.

To ensure the reliability of the validation process, we implemented a strict temporal window of 1095 days (three years) between the date of surveillance in abattoir to the field diagnostic event. Although the dataset contains longitudinal records spanning from 2017 to 2026, this three-year constraint was established to mitigate the confounding effects of environmental factors and alternative infection sources over time [10].

It is important to note that while this rigorous criterion was essential for the statistical validation of the model’s performance, it does not limit the practical utility of the risk classification for holdings outside this interval. Rather, the 1095-day threshold was employed to ensure that the Positive and Negative Predictive Values (PPV and NPV) were derived from the most epidemiologically stable data points, thereby enhancing the precision of the model’s benchmark without disregarding the broader applicability of the risk scores in routine surveillance.

The validation was further stratified based on whether surveillance successfully identified at least one positive farm per sentinel route. This allowed us to assess the model’s ability to identify at-risk farms in the presence of a confirmed field infection.

To evaluate the comparative performance of the risk tiers, the Positive and Negative Predictive Values (PPV and NPV) were used to calculate the Relative Risk (RR) across the different categories. To account for the limited size of the validated sample, the respective 95% confidence intervals (CI) were estimated using the Wilson score interval method. This approach was selected for its superior performance in maintaining coverage probability when dealing with small sample sizes or proportions near the boundaries of the distribution, ensuring a more reliable statistical inference. By comparing the risk classes through this framework, we quantified the incremental likelihood of identifying a field outbreak as a function of the model’s estimated probability.

To evaluate the practical utility of the prioritization model for surveillance resource optimization and policy strengthening, a retrospective simulation was conducted to quantify potential field inspection savings by applying the model prioritization.

### 2.4. Resources

All statistical analyses and modeling procedures were performed using Python (version 3.14.2) within Visual Studio Code (version 1.104.0) using the Jupyter extension (version 2026.1.0). Data manipulation and processing were conducted using Pandas (version 2.3.0) and DuckDB (version 1.2.0) libraries, with data stored in Apache Parquet format. Numerical processing and simulations were implemented with NumPy (version 2.1.2) and SciPy (version 1.15.0), while statistical validation and confidence interval estimations utilized Statsmodels (version 0.15.0) and SciPy.stats. To ensure scientific reproducibility, the complete source code and processed datasets are hosted in a public GitHub repository, as detailed in the Supplementary Materials section.

## 3. Results

**Figure 1:**
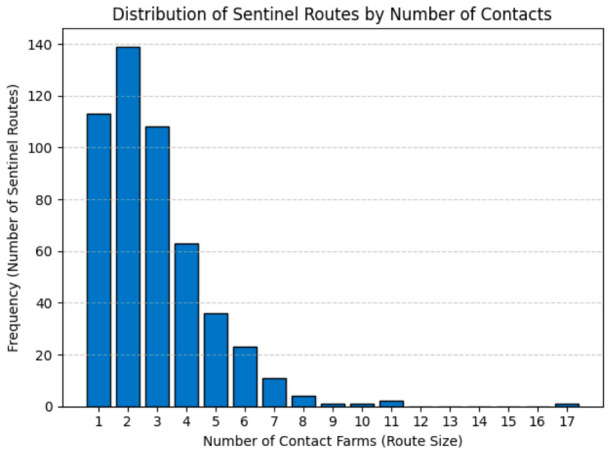
Distribution of Sentinel routes by number of contact farms.

Between 2021 and 2025, a total of 799 suspicious granulomatous lesions identified during routine abattoir inspections in 62 slaughterhouses across Santa Catarina state were included in this work. Following PCR analysis, 502 cases (62.8%) were confirmed as *Mycobacterium bovis* infections. These index cases were linked to a historical movement network comprising 1452 contact farms, while negative cases accounted for 844 associated farms. Within this cohort, 113 sentinel animals had a single movement record, having remained on only one farm throughout their lifetime prior to slaughter. A histogram showing the frequency of sentinel routes by the number of contact farms is presented in Figure 1.

The descriptive statistics for the primary variables incorporated into the risk model are summarized in Table 1. The livestock movement network analyzed through slaughterhouse surveillance involved 1452 sentinel–farm interactions. The data revealed a high degree of heterogeneity in population density and contact intervals. Herd sizes (H) exhibited a wide range, from 1 to over 45,000 animals, with a substantial difference between the mean (1339.2) and the median (171.7), indicating a distribution characterized by many small holdings alongside fewer large farms. Exposure duration (t) followed a similar pattern of high variability, with a mean contact time of 888.1 days (SD = 1185.6) and a median of 379 days. The sentinel animals visited, on average, 4.05 different farms during their lifetime.

The Monte Carlo simulation, comprising 5000 iterations for each contact, demonstrated high stability in the risk estimates. Across the historical movement network, 1452 contacts were identified, of which 1301 (89.6%) presented a calculable, non-zero infection probability (p > 0). The remaining 10.4% of contacts resulted in a null risk due to negligible exposure duration or herd size. Table 2 summarizes the stability metrics for these active interactions. By propagating the uncertainty of the daily infectious pressure (λ) through the PERT distribution, the resulting Coefficient of Variation (CV) of P remained consistently low, with a mean of 0.347 (±0.03) and a maximum value of 0.374. Furthermore, a sensitivity analysis of the risk classification (Table 3) showed a mean retention rate of 93.15% and a mean Cohen’s Kappa of 0.8773, indicating strong agreement between the stochastic and deterministic approaches. This narrow range of CV values and high tier stability confirm that the model’s output is highly robust to stochastic fluctuations, with risk variance being predominantly driven by the deterministic components of exposure (duration and herd size). Consequently, these results justified the use of the deterministic mean for the subsequent risk prioritization and field validation stages.

**Figure 2:**
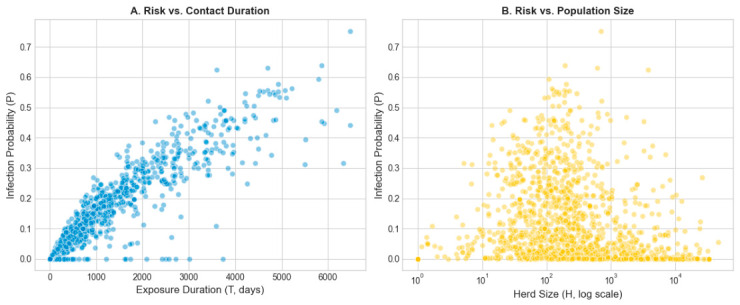
Scatter plots of infection probability (P) using the mean lambda versus exposure duration (T) (**A**) and herd size (H) (**B**).

The distribution of infection probabilities (P) as a function of the independent variables reveals the dynamic interaction between exposure duration and population density (Figure 2). The scatter plot of P versus exposure duration (T) shows a strong positive monotonic relationship, where risk increases progressively with the time the sentinel remained on the farm (Figure 2A). A higher concentration of elevated risk scores (p > 0.5) is observed in prolonged contacts; however, the significant vertical dispersion indicates that similar exposure times can result in markedly different risks depending on the herd size. Conversely, the relationship between P and herd size (H) displays a more heterogeneous dispersion pattern (Figure 2B). There is a noticeable density of high-risk scores in intermediate-sized herds (between 10^2^ and 10^3^ animals), resulting in a parabolic visual profile within the point cloud. The high vertical dispersion for any given value of H confirms that exposure duration acts as the primary risk modulator, capable of driving high infection probabilities even in smaller holdings or maintaining low risk in large herds if the contact period is brief.

As previously established, the 1452 movement contacts were stratified into three risk categories (Table 4). The High-Risk stratum concentrated 52.07% of the total contacts and exhibited a disproportionately higher average probability (p = 0.2057) compared to the other tiers. Notably, the Mean P  of the High-Risk group was nearly ten times greater than that of the Medium-Risk class and over 300 times higher than the Low-Risk baseline (p = 0.0006). A clear probabilistic gap was observed between the groups, as the minimum value for the High-Risk category (0.0518) remained strictly above the maximum value of the Medium-Risk tier (0.0513), ensuring a non-overlapping segregation of the data.

Of the 502 abattoir cases, 453 (90.24%) sentinel routes were subject to at least one field exam validated according to the described guidelines. The number of contact farms validated with field exams is shown in Table 5. The High-Risk category represented the largest share of the validated contacts, accounting for 58.00% (*n* = 319) of the total field effort. The remaining examinations were distributed between the Medium- (19.45%, *n* = 124) and Low-risk (22.54%, *n* = 107) classes. This distribution reflects the concentration of surveillance priority within the High-Risk stratum, which encompassed more than half of all holdings subjected to field diagnostic follow-up.

The model’s prospective predictive performance is summarized in Table 6. In the Global Performance analysis (*n* = 550), the High-Risk class achieved a PPV of 26.02% and an RR of 3.48 (95% CI: 1.33–5.81) compared to the Low-Risk reference (PPV: 7.48%). For this timeframe, the baseline Global Surveillance rate was 19.09%. Within the Surveillance Success subset, the High-Risk PPV increased to 82.18%, representing an RR of 2.57 (95% CI: 1.08–3.82) over the Low-Risk class (PPV: 32.00%). The baseline detection proportion in this scenario was 67.74%. Regarding the Negative Predictive Value (NPV), the Low-Risk class yielded 92.52% in the global analysis and 68.00% in the surveillance success subset.

Furthermore, the retrospective simulation demonstrated significant operational gains through risk-based prioritization. In the analysis of 389 traceback routes containing multiple contacts, the model’s prioritization strategy required a total of 1137 farm inspections compared to the 1339 visits conducted under the standard exhaustive protocol. By mandating the investigation of all high-risk farms while applying a sequential stopping rule for medium- and low-risk tiers, the model spared 202 unnecessary field inspections, representing a 15.09% reduction in the total surveillance workload without compromising the identification of positive cases.

## 4. Discussion

The integration of laboratory confirmation for granulomatous lesions is a fundamental component of bovine tuberculosis (bTB) surveillance in several countries, providing the empirical basis for monitoring disease-free status, estimating prevalence, and detecting emerging outbreaks [20,21,22,23,24,25,26].

Although the analytical sensitivity of individual tests can be limited by human factors such as the experience of inspectors [10,23,27,28] and animal factors related to the process of the formation of bovine tuberculosis visible lesions [29,30,31], the high frequency of slaughterhouse throughput enhances the aggregate sensitivity of this component, as the repeated screening of animals from the same origins increases the probability of detection over time [32,33,34].

From an operational perspective, our study emphasizes the impact of laboratory confirmation on the allocation of veterinary resources. The observed Positive Predictive Value (PPV) of 62.8% for laboratory-confirmed lesions is particularly significant for optimizing surveillance logistics. By ensuring that field investigations are triggered by confirmed cases, the official veterinary service minimizes the deployment of resources to holdings with a low probability of infection, thereby reducing the burden of inconclusive epidemiological inquiries. In this specific analysis, we found that the laboratory confirmation avoided 36.7% (844/2296) of the contact farms that could be subject of field inspections.

The structural complexity of the cattle movement network in Santa Catarina imposes a substantial epidemiological workload on the official veterinary service. Our findings confirm that the trace-back process for a single PCR-confirmed case is rarely a direct investigation of a single source; rather, it typically involves a complex chain of multiple contact holdings. With a mean and median of four contact farms per sentinel route, the discovery of a slaughterhouse signal necessitates the simultaneous management of several investigative fronts; frequently involving multiple cities and, consequently, different official state veterinary personnel.

The consistent route size observed throughout the dataset suggest that the need for multi-holding investigations is an inherent structural feature of the state’s livestock production system. From the animal health surveillance standpoint, this high connectivity creates an operational bottleneck, field veterinarians must rely on biological plausibility, such as dismissing contacts shorter than 30 days. Yet in the high-connectivity scenario of Santa Catarina, such rules of thumb may still leave a substantial number of farms to be audited. Without a standardized risk-ranking framework, the decision-making process for these investigations remains subjective, potentially leading to the dilution of surveillance efforts across contacts with varying degrees of risk.

The transmission dynamics of bovine tuberculosis are inherently stochastic and highly variable, yet as a chronic disease with slow progression, the probability of infection is fundamentally governed by temporal accumulation [35,36,37]. Our findings (Figure 2A) corroborate the premise that cumulative risk follows saturation curve rather than an indefinite linear progression, where the likelihood of transmission increases with exposure duration. This behavior is consistent with a non-homogeneous Poisson process framework and aligns with observations by others authors, who emphasize that while *M. bovis* transmission is relatively inefficient per contact event, the persistence of the pathogen and prolonged co-location in shared environments are the primary drivers of infection establishment [15,16,33]. While age is frequently categorized as a discrete risk factor [7,14], our model treats total exposure time as a continuous variable. By equating exposure to the animal’s life history, we achieve a more precise estimation of infection probability, capturing the temporal accumulation of risk that categorical proxies often overlook [24].

Furthermore, the influence of population density on infection risk revealed a biologically significant saturation pattern. Although larger herds (*H*) theoretically provide a denser network of potential transmission interfaces, our data (Figure 2B) indicate that this phenomenon is highly variable. The logarithmic transformation of *H* implemented in the model is justified by the observation that the marginal increase in risk diminishes as herd size grows, preventing the overestimation of infectious pressure in large-scale operations. This finding reflects established epidemiological theories suggesting that in large populations, contact structures tend to fragment into sub-groups, limiting the global infectious pressure exerted on an individual sentinel animal [38,39,40,41,42].

However, better data with comprehensive variables such as production type and herd management would probably be a better proxy of this effect [7]. It’s interesting to note that the divergence between mean and median values in Table 1 underscores a concentration of cattle in a minority of large holdings. This asymmetric distribution corroborates Veloso [7] and typifies the livestock sector in Santa Catarina: a high density of small-scale producers contrasted by a few high-capacity farming units.

The dominance of exposure duration and population density in driving risk variance (Table 2) justifies a deterministic simplification for broader use. By adopting the mean value of the infectious pressure (λ), the algorithm’s complexity was reduced without compromising its discriminatory power as shown in the sensitivity analysis (Table 3). This transition facilitates the integration of the framework as a decision-support tool within animal health databases.

Data presented in Table 4 demonstrate a complete absence of overlap between the High- and Low-Risk categories. This clear stratification validates the model’s discriminatory power and underscores its robustness as a reliable framework for risk classification.

The distribution of field investigations across risk strata suggests a high degree of convergence between the model’s epidemiological premises and the current expertise of official veterinarians. As shown in Table 5, most validated investigations (58%) were concentrated in holdings classified as High-Risk, indicating that the official service already prioritizes contacts with longer exposure durations and higher animal density. This alignment reinforces the model’s validity, as it formalizes the implicit criteria used by field staff into a standardized, quantitative framework. Rather than replacing veterinary judgment, the model serves to calibrate this intuition, providing a mathematical baseline that ensures consistent prioritization across different regions and personnel. Notably, the retrospective simulation demonstrated that even within this expert-driven environment, adopting the model’s standardized stopping rules could have reduced the total diagnostic workload by 15.09% (202 inspections) without compromising the detection of positive cases.

This empirical synergy is further quantified by the model’s predictive accuracy metrics. The incremental likelihood observed across risk classes (Table 6) demonstrates the model’s efficacy in concentrating infection probability within high-priority strata. Notably, the baseline surveillance PPV (19.02%) aligns with detection rates reported in similar epidemiological assessments in Ireland [43], providing a robust benchmark for our findings. The nearly four-fold increase in RR (*RR* = 3.48) for the High-Risk category suggests that prioritizing these holdings can optimize the yield of field investigations. Furthermore, the increase in PPV within the surveillance success scenario, which rose from 26.02% to 82.18%, indicates that the model’s performance is better captured when accounting for the sensitivity constraints of routine field testing. Although the Medium-Risk category presents greater statistical uncertainty, as the Wilson Score interval overlaps the reference group, its maintenance is justified by the observed directional trend in the point estimates. In an operational context of limited resources, this three-tier classification provides a tentative framework for sequential prioritization. While the High-Risk group remains the only category with a statistically significant increase in risk, the Medium-Risk group, with a PPV of 48.28%, still offers a marginal gain over the Low-Risk baseline of 32.00%. Thus, the framework serves as a more targeted alternative to current surveillance, despite the wider intervals imposed by the limited sample size in the surveillance success subset.

Despite its parsimonious structure, the model fulfills its primary operational objective by providing a robust quantitative basis for prioritizing epidemiological investigations. The results demonstrate that the framework capably maps infection probability within complex movement chains, transitioning from the analysis of isolated routes to a comprehensive network perspective. Beyond its immediate application as a decision-support tool for official veterinary services, the risk metrics developed here can be integrated into more sophisticated modeling architectures. For instance, these infection probabilities can serve as weighted inputs for spatial–temporal propagation models or Social Network Analysis (SNA) [16,44,45,46], where the “infectious pressure” identified in this study can refine the estimation of transmission nodes across broader cattle trade networks.

It is important to acknowledge that non-registered cattle movements may introduce a degree of uncertainty into the model’s accuracy. However, the impact of such omissions is likely mitigated by the high frequency of on-farm clinical inspections conducted by CIDASC; these routine visits serve to identify and reconcile discrepancies in movement records, as it is highly uncommon for a farm to remain uninspected for more than three years. Furthermore, while recording errors can impact model sensitivity, this effect is generally less pronounced for chronic diseases such as bovine tuberculosis compared to acute, highly infectious diseases like foot-and-mouth disease [47].

Furthermore, this reconciliation process is particularly relevant to the inclusion of short duration stays in the Lifetime Contact Networks. While the biological probability of infection during a very brief contact (e.g., <48 h) may be lower, short stays are more susceptible to recording errors than long-term residencies. For instance, a record indicating a one-day stay might represent a period of several months that was incorrectly logged or partially omitted. To account for these potential blind spots in movement data and to prioritize diagnostic sensitivity over specificity, no minimum threshold for duration of stay was applied. By including all recorded contacts, the model ensures that potential exposure events—which may be longer than the official record suggests—are not prematurely excluded from the risk classification.

Thus, the model provides both an immediate solution for field resource allocation and a modular foundation for future advancements in bovine tuberculosis surveillance and eradication strategies.

## 5. Conclusions

This study demonstrates that integrating quantitative risk estimation into slaughterhouse-triggered investigations can substantially enhance bovine tuberculosis surveillance in Santa Catarina. By combining official movement records with a probabilistic model based on exposure duration and time-weighted herd size, we generated biologically coherent infection-probability scores for each sentinel animal–farm interaction. The resulting three-tier risk classification effectively concentrated the likelihood of detecting true field infections within the High-Risk stratum, offering a significant improvement over the non-prioritized approach currently used in routine veterinary investigations.

The validation of this framework against field diagnostic outcomes showed that High-Risk holdings consistently exhibited markedly higher positive predictive values than Medium- or Low-Risk farms, indicating that the model captures the epidemiological features most strongly associated with transmission. Although medium-tier predictions carry greater uncertainty, the overall gradient of predictive performance supports the practical use of a stratified prioritization system.

However, the implementation of this prioritization tool is strictly dependent on the existence of a robust individual cattle tracking system. Fortunately, Brazil has established a strategic plan to implement individual traceability for the entire national cattle herd by the year 2032 [48], providing the necessary data infrastructure for the large-scale adoption of such risk-based models. By providing a standardized, transparent, and easily implementable tool, this approach can help official veterinary services allocate limited field resources more efficiently, reduce the frequency of inconclusive investigations, and accelerate outbreak detection. Beyond its immediate operational value, the model establishes a foundation for integrating risk-based metrics into broader network- and spatial-analysis frameworks, supporting long-term eradication strategies for bovine tuberculosis in the region.

## Figures and Tables

**Table 1:** Descriptive statistics of primary variables.

Variable	Mean	SD	Min	Q1	Median	Q3	Max
Herd size (*H*)	1339.25	3909.79	1.00	43.11	171.71	601.95	45,427.85
Exposure time (*t*)	888.07	1185.59	0.00	39.00	379.00	1290.25	6482.00
Route Size	4.05	2.48	1.00	2.00	4.00	5.00	17.00

**Table 2:** Stability Metrics of 5000 Monte Carlo simulations.

Contacts (P>0)	Mean CV	SD	Min	Q1	Median	Q3	Max
1301	0.347	0.03	0.184	0.332	0.359	0.37	0.374

**Table 3:** Sensitivity analysis of Risk Classification regards λ variation.

Metric	Value
Total Monte Carlo Iterations	5000
Mean Retention Rate (%)	93.15
Minimum Retention Rate (%)	84.09
Maximum Retention Rate (%)	95.80
Iterations with >90% Retention (%)	95.84
Mean Cohen’s Kappa	0.8773
Minimum Cohen’s Kappa	0.7319

**Table 4:** Risk classification summary of contact farms.

Risk Class	N	Mean	Median	Min	Max	% Total
High	756	0.206	0.177	0.052	0.752	52.07
Medium	349	0.023	0.0221	0.003	0.051	24.04
Low	347	0.001	0.000	0.000	0.003	23.90

**Table 5:** Contact farms validated with field exams per risk class.

Risk Class	Contact Farms	%
High	319	58.000
Medium	124	19.455
Low	107	22.545
Surveillance (total)	550	100.000

**Table 6:** Model and surveillance predictive metrics.

	Global Performance (*n* = 550 *)			Surveillance Success (*n* = 155 **)		
Risk Class	PPV (%)	NPV (%)	RR [95% CI]	PPV (%)	NPV (%)	RR [95% CI]
High	26.02	73.98	3.48 [1.33–5.81]	82.18	17.82	2.57 [1.08–3.82]
Medium	11.29	88.71	1.51 [0.54–3.36]	48.28	51.72	1.51 [0.61–2.91]
Low	7.48	92.52	1.00 (Ref)	32.00	68.00	1.00 (Ref)
Surveillance	19.09	80.91	-	67.74	32.26	-

* Number of validated contacts with field exams. ** Number of validated contacts in sentinel routes where at least one contact farm was found positive at field.

## Data Availability

The data presented in this study are openly available in the public repository at: https://github.com/lfelipecl/btb-abt-trace (accessed on 5 April 2026). All datasets provided in the repository have been de-identified to ensure anonymity, in compliance with Brazilian data protection laws (Lei Geral de Proteção de Dados-LGPD).

## Supplementary Materials

The analytical framework, source code, and additional supporting documentation associated with this study can be accessed in the public repository at: https://github.com/lfelipecl/btb-abt-trace (accessed on 5 April 2026).
